# Audit of Anesthetic Equipment in Veterinary Clinics in Spain and Portugal

**DOI:** 10.3389/fvets.2020.592597

**Published:** 2020-12-21

**Authors:** Jose I. Redondo, Luis Domenech, Cristina Mateu, Alfon Bañeres, Amalia Martínez, Diana Lopes

**Affiliations:** ^1^Departamento de Medicina y Cirugía Animal, Facultad de Veterinaria, Universidad Cardenal Herrera-CEU, CEU Universities, Valencia, Spain; ^2^Departamento de Matemáticas, Física y Ciencias Tecnológicas, Escuela Superior de Enseñanzas Técnicas, Universidad Cardenal Herrera-CEU, CEU Universities, Valencia, Spain; ^3^Ecuphar Veterinaria SLU, Barcelona, Spain; ^4^Belphar Lda, Sintra, Portugal

**Keywords:** anesthesia, veterinary, equipment malfunction, safety, anesthetic machine, vaporizer, audit, retrospective study

## Abstract

The objective of this retrospective study was to review the results of a 4-year audit performed on anesthetic machines and vaporizers used in veterinary clinics in Spain and Portugal. Data was collected between July 2016 and April 2020. Inspections were carried out by a team of seven veterinarians, using a human-modified system of checks that was adapted to a veterinary practice. The evaluation of each item was noted as “correct” or “incorrect”. The vaporizers' performance was evaluated using a self-calibrating gas analyzer. The vaporizer was classified as “correct” or “incorrect” when the vaporization error was less than or equal to, or more than 20%, respectively. The anesthetic machine was classified as “conforming” if all its components were noted as “correct” and no leaks were detected, or as “non-conforming” if any of the components was noted as “incorrect” or if a leak was detected. If the inspector was able to repair on-site the item malfunctions detected and the machine was fit for use, they issued a final report as “conforming.” On the contrary, if such malfunctions persisted, the final report was “non-conforming,” and a recommendation to remove the machine from service until its final repair was provided. To perform statistical analysis, each inspected item was used as predictor, classification and regression trees were built, and a random forest analysis was performed. A total of 2,001 anesthetic machines and 2,309 vaporizers were studied. After inspection, 42.7 and 26.4% of the machines were non-conforming and conforming, respectively, whereas 30.9% could be repaired *in situ*. A total of 27.1% of the isoflurane vaporizers and 35.9% of the sevoflurane vaporizers were incorrect. Machine learning techniques showed that the most important variables in the classification of the anesthetic machines as conforming or non-conforming were mostly the scavenger system and the canister, followed some way behind by the APL valve, source of oxygen, reservoir bag, vaporizer, and connections.

## Introduction

Modern anesthesia workstations are an integration of several components required to safely administer anesthesia to a patient. They consist of the anesthetic machine, vaporizers, ventilator, breathing systems, scavenging system, and monitors. Medical gases (i.e., air, oxygen, nitrous, oxide) are supplied through central units or oxygen concentrators, pipeline systems, terminal units, and hoses that connect these to the anesthesia machine. Breathing systems connect the anesthetic machine to the patient and dispense a controlled composition of medical gas mixture (1). Although many current anesthetic workstations include numerous safety mechanisms such as safety self-checks or oxygen failure safety devices, older and simpler anesthetic machines are still in use in a considerable number of Spanish and Portuguese veterinary clinics.

It has been widely demonstrated in human medicine that the malfunctioning of anesthetic equipment can lead to severe complications and fatal outcomes (2–5). Some reports on equipment failure resulting in complications have also been published in veterinary medicine (6–8). Interestingly, and although human safety guidelines establish protocols for routine inspection of anesthetic machines (9–11), a research study performed in France concluded that most equipment malfunctions were due to lack of routine maintenance (3).

Unlike in human medicine, veterinary clinics in Spain and Portugal are not obliged to follow safety protocols in regard to the anesthetic equipment, and these remain mere recommendations for a safe practice. To the Authors' knowledge, the only audit of veterinary anesthetic equipment to date was performed in New Zealand, it was published in 1995, and detected that 91% of anesthetic machines were faulty (12). Therefore, the objective of this retrospective study was to review the results of a 4-year audit performed on anesthetic machines and vaporizers used in veterinary clinics in Spain and Portugal, that had a contract with two pharmaceutical companies.

## Materials and Methods

Anesthetic machines, vaporizers and other anesthetic equipment from veterinary clinics that had a commercial relationship with two veterinary pharmaceutical companies (i.e., Ecuphar Veterinaria in Spain and Belphar Lda. in Portugal) and that were inspected between July 2016 and April 2020 were analyzed in this report. Data collection included date of inspection, name and address of the clinic, and the name of the veterinarians who commonly used the anesthetic equipment. However, this data was partially anonymized for privacy, in accordance with the General Data Protection Regulation EU 2016/679 (13), and only date and province in which the inspection was performed was noted.

Inspections were carried out by a team of seven veterinarians experienced in clinical anesthesia, employees of the aforementioned companies, who had additionally received a specialized training in anesthetic equipment. The inspection team developed its own checking-up protocol based on the guidelines of the Sociedad Española de Anestesiología y Reanimación (14) and adapted it to a standard veterinary practice.

Firstly, the anesthetic machine was inspected, beginning with a visual evaluation that consisted of noting its brand and model, absence or presence of components, state of conservation, location within the clinic and marks of previous revisions. Additionally, the main user of the machine was asked if leaks, component malfunctions, excessive consumption of oxygen or anesthetic agent, or any notable adverse events were detected during use.

Secondly, each item of the anesthetic machine was inspected and classified as “correct” or “incorrect.” Detected failures were noted and repaired on site, if possible, in which case the item was reclassified (Table 1). The type of oxygen source (i.e., cylinder or concentrator) available was noted, connected, and checked. The oxygen concentration provided by the concentrator was evaluated using a Dräger VAMOS anesthetic gas analyzer (Drägerwerk AG & Co. KGaA, Lübeck, Germany). Connections, pressure gauges and pressure reducing valves, flowmeters, adjustable pressure limiting (APL) valve, and oxygen emergency valve were inspected. The canister and the soda lime were visually inspected. Then, a leak test was performed. The corrugated tubes and the reservoir bag were attached correspondingly, another reservoir bag was placed at the connection to the endotracheal tube, and the APL valve was closed. Then, a fresh gas flow of 1–2 L/min was provided until the pressure gauge of the system indicated 30 cm H_2_O. Then, the flow was interrupted, and the pressure gauge visualized to ensure the pressure was maintained. In the case of a decrease in pressure, the flow required to maintain 30 cm of H_2_O of pressure was recorded to quantify the leak and this number noted as the total leakage of the system. In accordance to standard recommendation (15), a maximum of 0.3 L/min was considered acceptable. Location of the leak involved the hearing of an audible sound or the use of a leak detection spray, and these were fixed if possible. Availability of reservoir bags in different sizes and their quantity was also noted and finally, other anesthesia breathing systems (i.e., Mapleson A, D, and E) were also inspected.

**Table 1:** List of items inspected, possible failures detected during inspection, their classification as repairable *in-situ* and by which method they could be repaired.

**Item**	**Failure**	**Repairable**	**Repair method**
Oxygen source: cylinder	Improperly installed or inspection expired	Yes (if spare parts are available)	Replace
	Leaking connections	Yes	Repair
	Absence of safety clamps	Yes	Add
	Inadvertently opened	Yes	Close
Oxygen source: concentrator	Presence of dust in external filters	Yes	Clean
	Low oxygen purity (<50% not acceptable, 51–80% check, >81% acceptable)	No	Technical service
	Maximal flow delivered <5 L/min	No	Technical service
	Improper attachment of the humidifier	Yes	Remove
Oxygen hose	Leakage due to a crack, breakage or instability in silicone or plastic hoses, elbows and joints.	Yes	Repair
Connections	Leakage due to cracks or breaks	Yes (sometimes)	Repair
	Leaks due to incorrect fitting and lack of watertightness	Yes (sometimes)	Repair
Pressure gauges	Absence	No	Replace
	Malfunction	No	Repair
	Not calibrated to zero	Yes (sometimes)	Repair
	Absence of protective cap	No	Replace
Flowmeter	Absence	No	Replace
	Tilted or unstable	Yes (sometimes)	Repair
	Obstructed or faulty	No	Replace
Vaporizer	Decalibrated (under- or over-vaporization)	Yes (not immediately)	Technical service
	Leakage at the point of connection to the unit (missing O-rings on Selectatec block, incorrectly fitted, locking mechanism in open position)	Yes	Repair
	Leaks in the filling system (key-fill system left open, anesthetic liquid dripping from poorly-tightened screw-fill base)	Yes	Adjust
	Non-reconverted halothane vaporizer, in use with isoflurane	Yes (but not immediately)	Reconversion
	Deteriorated or stiff dial	No	Technical service
Oxygen valve	Absence	No	Replace
	Faulty	No	Repair/Replace
	Malfunction	No	Repair/Replace
Fresh gas outlet	Leakage in connecting parts	Yes (sometimes)	Repair
	Badly threaded outlet part	Yes (sometimes)	Repair
Inspiratory valve	Absence	No	Replace
	Not properly closed (slight leakage)	Yes	Adjust
	Broken dome or base (heavy leakage)	No	Replace
	Dirt	Yes	Clean
Expiratory valve	Absence	No	Replace
	Not properly closed (negligible leakage)	Yes (sometimes)	Adjust
	Broken dome or base (non-negligible leakage)	No	Replace
	Dirt	Yes	Clean
	Excess humidity	Yes	Clean
Negative pressure valve	Malfunction	No	Repair
APL valve	Absence	No	Replace
	Malfunction (opens at pressure <20 cm H_2_O)	No	Repair/Replace
	Faulty (does not open even if in open position, thereby causing overpressure in reservoir bag and airway).	No	Repair/Replace
	Cracks or fissures	No	Repair/Replace
Canister	Saturated soda lime	Yes	Replace
	Excess or shortage of soda lime	Yes	Adjust
	Leakage due to lack of O-rings at the base or top	Yes (if spare parts are available)	Repair
	Leakage due to dirt or soda lime in the junctions	Yes	Clean
	Leakage due to cracks or fissures (heavy leakage)	No	Replace
Corrugated tubes	Deteriorated (dirt, fungus, moisture)	No	Replace
	Cracked or broken	No	Replace
	Lack of availability of several sizes (at least two between the neonatal, pediatric and adult units)	No	Replace
Reservoir bag	Perforated	Yes (if spare parts are available)	Replace
	Lack of availability of several sizes (a range of sizes between 0.5, 1, 2, 3, 4 L are advisable)	No	Replace
Mapleson A	Deteriorated (dirt, fungus, moisture)	No	Replace
	Cracked or broken	No	Replace
Mapleson D	Fitted the wrong way (inspiratory branch in the expiratory part and vice versa)	Yes	Change position
	Deteriorated (dirt, fungus, moisture)	No	Replace
	Cracked or broken	No	Replace
Mapleson E	Absence	No	Replace
	Open bag system, no APL valve or waste gas collection	Yes (if spare parts are available)	Install valve and a closed reservoir bag
	Cracked or broken	No	Replace
Scavenger system	Missing device	Yes	Install
	Collection only post-APL but not post-ventilator	Yes	Install
	Active absorption system malfunction	No	Technical service
	Saturated activated charcoal	Yes (if spare parts are available)	Replace

Thirdly, the type of scavenging system was noted, and each item evaluated (Table 1). If passive systems (activated charcoal canisters) were used, they were weighed to determine their degree of saturation. If an anesthetic gas scavenging system (AGSS) was in use, the suction rate was checked.

Then, vaporizers' performance was evaluated using a Dräger VAMOS anesthetic gas analyzer (Drägerwerk AG & Co. KGaA, Lübeck, Germany). This analyzer, which has a self-calibration system, has a measurement range of 0–8.5% for isoflurane and 0–10% for sevoflurane, with a precision of one decimal place and an anesthetic agent accuracy ±(0.2 vol % + 15% relative). In addition, this analyzer was periodically calibrated following manufacturer's recommendations. The inhalant anesthetic agent used in the agent-specific vaporizers, whether isoflurane or sevoflurane, was noted. A system that consisted of an airway adapter, a filter, and a sampling line connected to the gas analyzer was used for sidestream analysis. Depending on the machine analyzed, the inspector selected the location where to take the sample from (i.e., the vaporizer outlet, the fresh gas outlet, or the Y piece). The measured vaporization (M) displayed on the analyzer was compared to three selected vaporization percentages (i.e., 0.5, 2, and 3%) on the vaporizer dial (V) at four different fresh gas flows (i.e., 0.5, 1, 2, and 3 L/min). The vaporization error (E) was calculated as E = (M – V)/V. The vaporizer was classified as “correct” or “incorrect” when E was less than or equal to, or more than 20%, respectively (16). The vaporization data obtained at 0.5% were recorded but excluded from the final classification of the machine.

The anesthetic machine was classified as “conforming” if all its components were noted as “correct” and no leaks were detected, or as “non-conforming” if any of the components was noted as “incorrect” or if a leak was detected. If the inspector was able to repair on-site the item malfunctions detected and the machine was fit for use, they issued a final report as “conforming.” On the contrary, if such malfunctions persisted, the final report was “non-conforming,” and a recommendation to remove the machine from service until its final repair was provided.

### Statistical Analysis

The statistical language R 4.0.2 was used. Firstly, a descriptive study of data was carried out. Parametric variables are shown as mean ± standard deviation. Non-parametric numerical variables are shown as median and interquartile range. Qualitative variables are expressed as number of observations and frequency tables. Secondly, machine learning analysis, such as classification and regression trees and random forest analysis were performed to study the outcome of the inspection (conforming/non-conforming), using the result of the inspection of each part of the anesthetic machine (correct/incorrect) as predictors.

## Results

**Figure 1:**
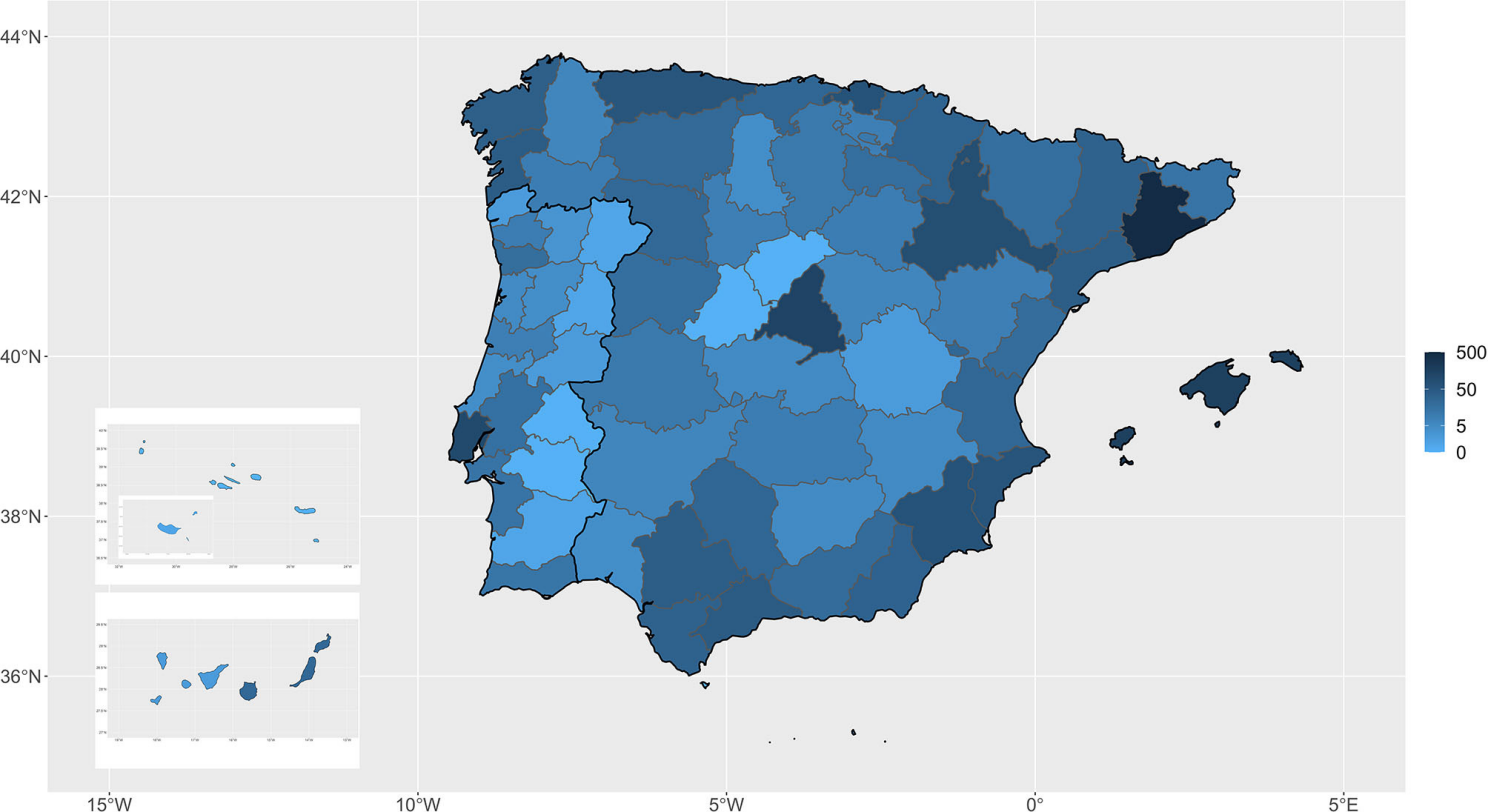
Distribution of the anesthetic machines that were audited in Spain and Portugal.

A total of 573 veterinary clinics from Spain and 119 from Portugal participated in this study, and 2,001 anesthetic machines and 2,309 vaporizers were inspected (Figure 1). The median number of the machines studied by province was 11. The provinces in which most inspections were made were Barcelona (438), Madrid (139), the Balearic Islands (162), and Lisbon (105). Only three Spanish provinces (Avila, Segovia and Melilla) and three Portuguese provinces (Azores, Evora, and Portalegre) had no inspections performed.

**Figure 2:**
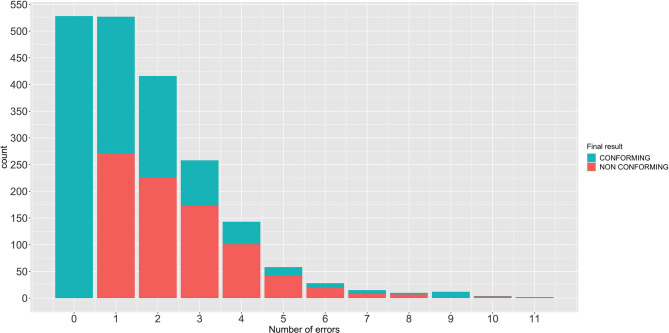
Number of anesthetic machines repaired separated by the number of malfunctions found and their final classification as conforming or non-conforming.

**Figure 3:**
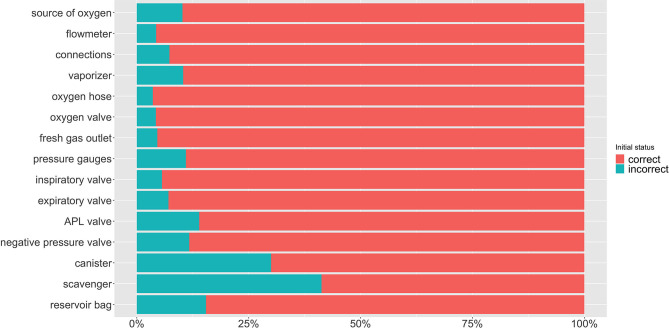
Percentage of malfunctions detected on each component of the anesthetic machine at initial inspection and their classification as correct or incorrect.

At initial evaluation, 528 machines (26.4%) and 1,473 (73.6%) were classified as conforming and non-conforming, respectively. The median (range) of incorrect items detected in non-conforming machines was 2 (1–11). One incorrect item was detected in 527 machines, 2 in 416, 3 in 258, 4 in 143, 5 in 58, and 6 or more in 71. Out of the 1,473 machines initially classified as non-conforming, the inspector was able to repair 619 machines (30.9%), which were therefore reclassified as conforming (Figure 2). In summary, at the end of the inspection, 53.3% of the machines were found to be conforming, while the remaining 42.7% were recommended that they were withdrawn from service until further repair. The percentages of malfunctions detected in the machine components are shown in Table 2 and Figure 3.

**Table 2:** Percentage and number of malfunctions after the initial inspection of the different parts of the anesthetic machine, circuits, and scavenger.

**Item**	**Correct**	**Incorrect**	**% of malfunction**
Oxygen source	1800	201	10.0
cylinder	476	9	1.9
concentrator	1226	181	12.9
Oxygen hose	1938	63	3.1
Connections	1862	139	6.9
Pressure gauge	1808	193	9.6
Flowmeter	1922	79	3.9
Vaporizer	1656	653	28.3
isoflurane	1454	540	27.1
sevoflurane	202	113	35.9
Oxygen emergency valve	1931	70	3.5
Fresh gas output	1916	85	4.2
Inspiratory valve	1909	92	4.6
Expiratory valve	1884	117	5.8
Corrugated tubes	1858	143	7.1
APL valve	1777	224	11.2
Negative pressure valve	1949	52	2.6
Canister	1498	503	25.1
Reservoir bags	1774	227	11.3
Mapleson E system	771	211	21.5
Mapleson D system	334	18	5.1
Mapleson A system	642	75	10.5
Scavenger	1243	758	37.9

Out of the 2,001 machines, 1,407 used a concentrator as oxygen source, 485 an oxygen cylinder and 109 lack of an oxygen source, which were mobile secondary equipment. Most of the clinics used activated charcoal canisters as scavenger system and only seven veterinary clinics had an AGSS.

**Figure 4:**
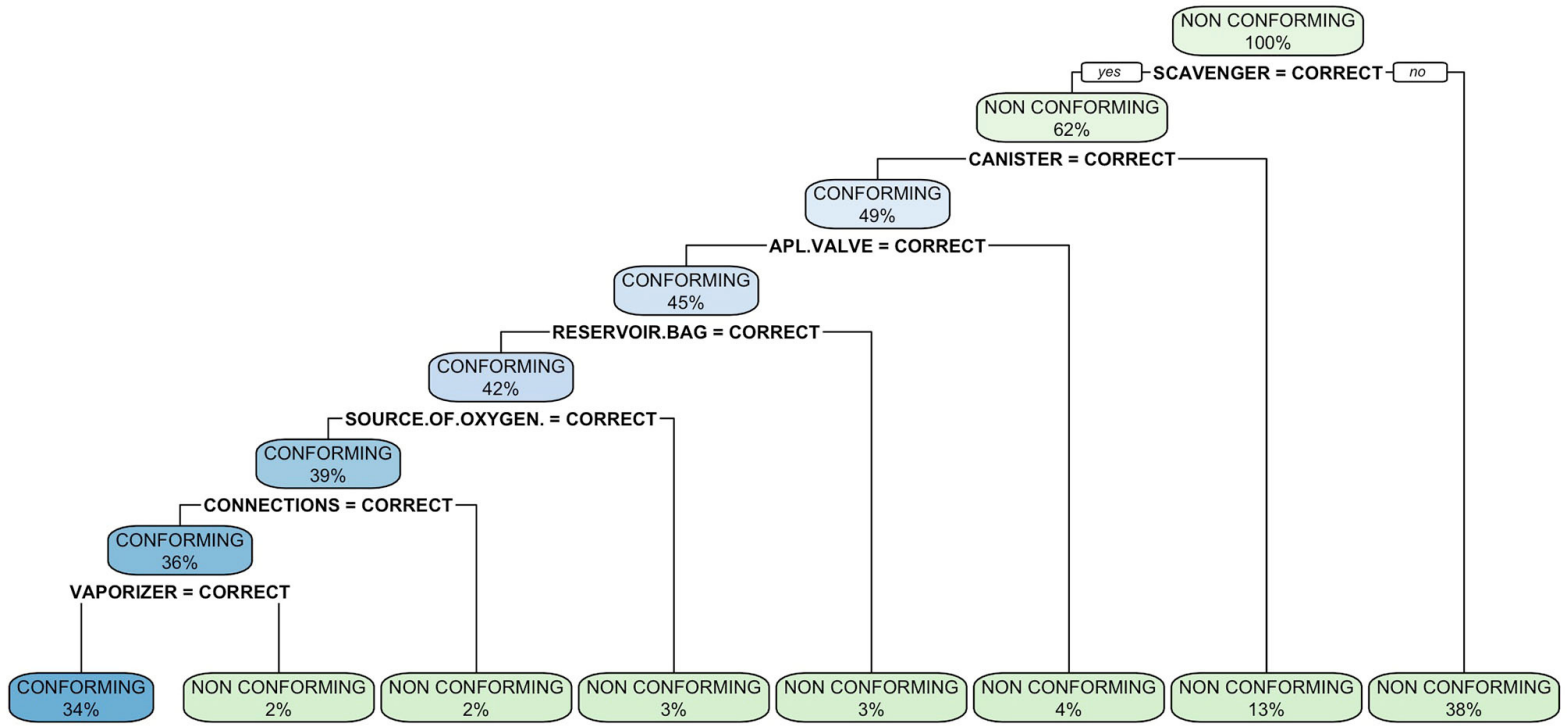
Classification tree of malfunctions detected at inspection of anesthetic machines. The classification tree represents the different selection criteria or “decision nodes” used to predict the most correct classification of the total number of cases (represented at the root of the tree as a 100%). As the data is classified in subsets, the percentage value represents the probability of a case of belonging to that data subset.

**Figure 5:**
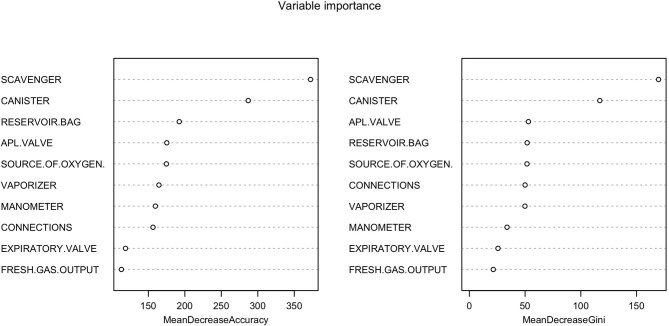
Random forest showing the importance of each variable in the inspection of anesthetic machines. The Mean Decrease Accuracy plot expresses how much accuracy the model losses by excluding each variable. The more the accuracy suffers, the more important the variable is for the successful classification. The variables are presented from descending importance. The mean decrease in Gini coefficient is a measure of how each variable contributes to the homogeneity of the nodes and leaves in the resulting random forest. The higher the value of mean decrease accuracy or mean decrease Gini score, the higher the importance of the variable in the model.

A total of 2,309 vaporizers were revised, 1,994 of which used isoflurane and 315 sevoflurane. In total, 1,656 were found to be correct while the remaining 653 were found to be incorrect (Tables 3, 4 and Figures 4, 5).

**Table 3:** Percentage of isoflurane vaporizers that malfunction according to the position of the dial and the gas flow used.

	**Vaporizer 0.5%**				**Vaporizer 2%**				**Vaporizer 3%**			
**Error**	**0.5 L/min**	**1 L/min**	**2 L/min**	**3 L/min**	**0.5L/min**	**1 L/min**	**2 L/min**	**3 L/min**	**0.5 L/min**	**1 L/min**	**2 L/min**	**3 L/min**
< (−20)%	2.7	2.5	2.5	2.8	2.6	1.6	1.4	1.5	2.1	1.4	1.6	1.5
(−20) to (−10)%	10.2	8.8	9.6	10.4	5.0	3.9	2.8	2.7	3.1	2.3	1.8	1.9
(−10) to 0%	0	0	0	0	20.5	17.1	15.6	15.7	19.7	17.0	14.0	14.5
0%	36.7	34.7	34.7	35.6	20.8	19.9	17.8	20.9	13.8	11.1	11.9	13.3
0–10%	0	0	0	0	29.6	32.5	34.8	35.0	36.5	39.4	39.4	41.4
10–20%	36.7	39.8	39.4	38.4	14.5	16.6	19.0	17.1	17.9	21.3	23.5	21.2
<20%	13.7	14.2	13.8	12.8	7.0	8.4	8.6	7.1	6.9	7.5	7.8	6.2

*L/min, liters per minute*.

**Table 4:** Percentage of sevoflurane vaporizers that malfunction according to the position of the dial and the gas flow used.

	**Vaporizer 0.5%**				**Vaporizer 2%**				**Vaporizer 3%**			
**Error**	**0.5 L/min**	**1 L/min**	**2 L/min**	**3 L/min**	**0.5 L/min**	**1 L/min**	**2 L/min**	**3 L/min**	**0.5 L/min**	**1 L/min**	**2 L/min**	**3 L/min**
< (−20)%	3.9	3.2	2.3	2.7	3.9	2.9	1.9	1.7	3.5	2.3	2.3	1.7
(−20) to (−10)%	12.9	11.6	12.2	10.7	8.4	6.4	5.2	5.0	6.4	6.5	5.5	3.3
(−10) to 0%	0	0	0	0	19.6	16.4	13.7	13.0	24.8	19.7	17.6	18.0
0%	29.5	28.3	29.9	28.2	18.6	17.6	22.2	18.6	11.3	11.7	14.3	12.3
0–10%	0	0	0	0	28.2	34.0	30.9	31.7	33.4	36.2	35.5	37.3
10–20%	36.0	37.9	39.1	40.7	14.2	15.7	17.6	21.7	16.1	19.1	18.6	21.7
<20%	17.7	19.0	16.5	17.7	7.1	7.0	8.5	8.3	4.5	4.5	6.2	5.7

*L/min, liters per minute*.

**Figure 6:**
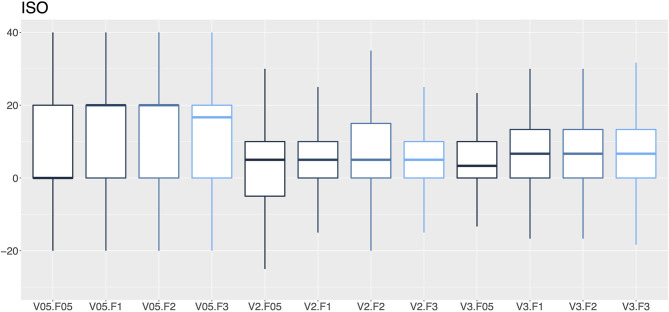
A box-and-whisker diagram (median, interquartile range, and extremes) of percentage errors as a function of the vaporization percentage (V; 0.5, 2 and 3%) and the gas flow (F; 0.5, 1, 2 and 3 L/min) for isoflurane vaporizers.

**Figure 7:**
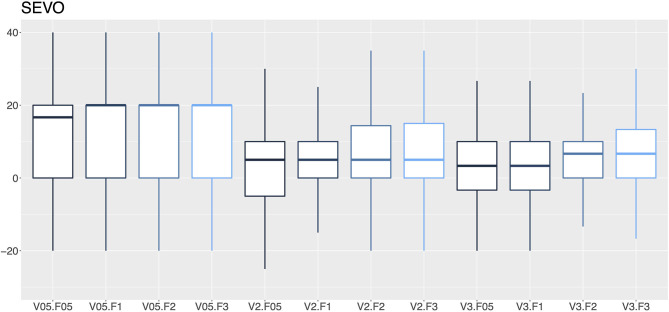
A box-and-whisker plot (median, interquartile range, and extremes) of percentage errors as a function of the vaporization percentage (V; 0.5, 2, and 3%) and gas flow (F; 0.5, 1, 2, and 3 L/min) for sevoflurane vaporizers.

The classification tree analysis showed that the most important variables in the classification of the anesthetic machines as either conforming or non-conforming were, in descendent order, the scavenger system, the canister, the APL valve, the reservoir bag, the oxygen source, connections and the vaporizer (Figure 6). In the random forest analysis, the greatest decrease of the Gini index was found with the scavenger system and the canister, followed some way behind by the APL valve, source of oxygen, reservoir bag, vaporizer, connections, manometer, expiratory valve, and fresh gas output (Figure 7). The random forest algorithm correctly classified 100 and 96.1% of the conforming and non-conforming anesthetic machines, respectively.

## Discussion

This audit detected that 73.6% of the anesthetic machines were non-conforming. During revision, the inspector was able to solve the malfunction *in-situ* in 30.9% of the machines. A total of 27.1% of isoflurane and 35.9% of sevoflurane vaporizers were found to be non-conforming. Machine learning techniques showed that the most important variables in the classification of the anesthetic machines as either conforming or non-conforming were mostly the scavenger system and the canister, followed by the oxygen source, the expiratory and APL valves, the reservoir bags and the vaporizers.

In a study of 64 anesthetic machines in veterinary clinics in New Zealand, the percentage of malfunctions was as high as 91% (12). In this audit 73.6% of the machines were initially classified as non-conforming. This result could be considered comparable to the 91% due to the small sample size it included. Although 25 years have passed between both studies, the advancement in technology and equipment doesn't seem to be translated to significantly better results in regard to proper maintenance of anesthetic equipment.

The use of anesthetic equipment checklists is recommended by most human and veterinary anesthesia clinical guidelines (9–11, 14). Several human studies state that most of the complications arising from equipment malfunction are due to an inadequate machine check, especially between cases as opposed to at the beginning of the day (17, 18). It should be noted, however, that the use of checklists should not be intended to replace regular inspections by trained personnel, but rather complement it. This audit did not record the use of or compliance with such checklists and therefore the impact that the use of these may pose on the functioning of the equipment cannot be assessed. Nevertheless, the results of this audit may serve to highlight the importance of the use of checklists since most of the malfunctions detected, which led to classify the anesthetic machines as not-conforming, could have been identified and prevented by the daily use of in-house checklists. Since the routine implementation of a checklist, such as the internationally approved Association of Veterinary Anesthetists (AVA) one (19), could have avoided some of most common malfunctions detected, further audits that include the use of preanesthetic checklist are warranted.

The classification tree and the random forest analysis agreed in that malfunctions were usually found in the scavenger system, the canister, the APL valve, the reservoir bag, the oxygen source, connections, and the vaporizer. The algorithms obtained thereof may help to optimize the inspection of anesthetic machines. The component in which most problems were observed was the scavenger system, 37.9% of which were found to be non-conforming. Most malfunctions were due to the absence of a scavenger system or an exhausted activated charcoal canister. Chronic exposure to an environment contaminated with inhalational anesthetics can cause chronic toxicity in exposed personnel (20, 21). When evacuation systems are used, no study has been able to demonstrate that traces of anesthetic gases negatively affect operating room personnel (18). In fact, Spanish regulations establish a limit value—daily exposure (VLA-ED) of isoflurane and N2O (22).

The second most frequent component to malfunction was the canister (25.1%). Canisters require regular inspections, as the absorbent contained within must be changed when it is exhausted. Improper sitting of the canister, accumulation of dust in the junctions, or malfunctioning of the absorbent may result in adverse events to the patient and personnel (1). This finding is in agreement with other studies stating that the canister could be one of the main sources of leaks in the anesthetic machine (23).

The reservoir bag was the third most frequent component to malfunction (11.3%). In veterinary medicine, reusable latex, silicone or rubber bags are normally used. Over time, when exposed to high oxygen concentrations, the material loses its elasticity and becomes damaged, especially in the area where the bag is attached to the anesthesia breathing system (24). Reservoir bags should be checked regularly and replaced if leaks are detected. Additionally, Inspectors frequently reported lack of availability of varied reservoir bag sizes which poses a risk of overinflation when a reservoir bag smaller than the indicated one is used (25).

The fourth component that most frequently malfunctioned was the APL valve (11.2%) and the failures detected could never be repaired *in situ* by the inspector. If the APL valve cannot be fully open, it may cause from a small increase in airway resistance to barotrauma. Conversely, if it cannot be fully closed, it may preclude positive pressure ventilation. This malfunction has been also frequently observed in humans (26).

The oxygen source failed in 10.0% of the machines. Malfunctions were recorded in only 1.9% of the cylinders, compared to 12.9% of the O_2_ concentrators. In recent years, the use of oxygen concentrators in veterinary anesthesia has become popular in Spain and Portugal due to their versatility and low cost and as an alternative to medicinal oxygen bottles. These devices concentrate ambient oxygen and provide FiO_2_ up to 95% (27). However, they do need regular maintenance and checking, especially of the O_2_ concentration and the fresh gas flow they provide. A concentrator malfunction can result in a hypoxic mixture, which can cause adverse effects to the patient, and even death (5).

The inspiratory and expiratory valves are two of the most important components of the circle system. Their malfunction can result in adverse effects such as rebreathing of expired air, respiratory collapse and barotrauma (1). Malfunctions were detected in 4.6 and 5.8% of the machines, respectively. Valves can break or become blocked by secretions or moisture and therefore, the availability of spare valves for each machine and regular inspection are recommended (28).

In this audit, 27.1% of the isoflurane vaporizers and 35.9% of the sevoflurane vaporizers had vaporization errors >20% with flows of 2 and 3 L/min and therefore required recalibration by a technical service. An error rate ranging from 12.8–14.2% to 16.5–19.0% in isoflurane and sevoflurane vaporizers was observed, respectively. The VAMOS anesthetic gas analyzer has an accuracy of one decimal place and overestimates the error at low percentages of vaporization, which could be considered a limitation of the study. For example, if the vaporizer dial is set to 0.5% and the actual vaporization percentage is 0.55%, the analyzer will display 0.6%, which represents an error of 20%. In this study, VAMOS was used because it can detect a 20% error. However, a more accurate analyzer would be required to evaluate the performance of the vaporizer at low percentages. In this audit, the minimal studied flow was 0.5 L/min which is the minimal flow recommended for accurate vaporization (29). Therefore, low-flow techniques, which are highly recommended to reduce contamination and consumption of anesthetics and oxygen (30), require the use of gas analyzers that provide in real time the anesthetic concentration administered to the patient (29). Any increase or decrease in the vaporization percentage of inhalational anesthetics can lead to adverse events such as anesthetic overdose, hypotension, decreased cardiac output, respiratory depression or even intraoperative awakening (31).

Our study has several limitations. Firstly, there is a sample bias. This study was not randomized, since only veterinary clients that had a commercial relationship with Ecuphar Veterinaria SLU in Spain or Belphar Lda in Portugal have been included. Based on a total count of 6,228 and between 1,400 and 1,600 veterinary clinics present in Spain and Portugal in 2019 (32), respectively, we can estimate that in this study more than 9.2% of the Spanish clinics and at least 7.4% of the Portuguese clinics were included. However, the large sample size of analyzed machines and vaporizers could mitigate this limitation. Secondly, this was a retrospective study based on a review of reports compiled by a technical team of seven people. Although the training and technical criteria of the team were similar, a subjective component when it comes to finding equipment malfunctions may have also taken place. Thirdly, neither the private data of the clinics nor the equipment inspected were identified during this audit in such a way to allow traceability and therefore, some machines and vaporizers may have been inspected several times in different years. Further studies that keep track of the inspected equipment would be required to determine the effect that regular inspections have on their functioning over time.

In this study, most audited machines malfunctioned during the inspection and the inspector was able to repair a significant number of them *in situ*. In conclusion, a regular revision of anesthetic equipment by qualified personnel and the daily implementation of routine checklists are key to ensure proper functioning and to avoid adverse effects on the patient, personnel, and environment.

## Data Availability Statement

The raw data supporting the conclusions of this article will be made available by the authors, without undue reservation.

## Author Contributions

JR and LD contributed to the conception of the study, interpretation, drafted and revised the manuscript, and approved the final version. CM, AB, AM, and DL contributed to the data acquisition, drafted and revised the manuscript, and approved the final version. All authors contributed to the article and approved the submitted version.

## Conflict of Interest

The authors declare that this study received funding from Ecuphar Veterinaria SLU and Belphar Lda. The funders were not involved in the study design, collection, analysis, interpretation of data, the writing of this article or the decision to submit it for publication. The handling editor declared a past co-authorship with one of the authors JR.
